# A Review of Direct Neck Measurement in Occupational Settings

**DOI:** 10.3390/s101210967

**Published:** 2010-12-03

**Authors:** Letícia Carnaz, Mariana V. Batistao, Helenice J. C. Gil Coury

**Affiliations:** Department of Physical Therapy, Universidade Federal de São Carlos, CP 676, CEP 13565-905, São Carlos, SP, Brazil; E-Mails: lecarnaz@gmail.com (L.C.); maribatistao@hotmail.com (M.V.B)

**Keywords:** portable equipment, direct measurements, cervical movement, occupational exposure

## Abstract

No guidelines are available to orient researchers on the availability and applications of equipment and sensors for recording precise neck movements in occupational settings. In this study reports on direct measurements of neck movements in the workplace were reviewed. Using relevant keywords two independent reviewers searched for eligible studies in the following databases: Cinahal, Cochrane, Embase, Lilacs, PubMed, MEDLINE, PEDro, Scopus and Web of Science. After applying the inclusion criteria, 13 articles on direct neck measurements in occupational settings were retrieved from among 33,666 initial titles. These studies were then methodologically evaluated according to their design characteristics, exposure and outcome assessment, and statistical analysis. The results showed that in most of the studies the three axes of neck movement (flexion-extension, lateral flexion and rotation) were not simultaneously recorded. Deficiencies in available equipment explain this flaw, demonstrating that sensors and systems need to be improved so that a true understanding of real occupational exposure can be achieved. Further studies are also needed to assess neck movement in those who perform heavy-duty work, such as nurses and electricians, since no report about such jobs was identified.

## 1. Introduction

Work-related neck disorders are associated with a high degree of pain and incapacitation [1]. This fact can be demonstrated by the high prevalence of neck pain and related musculoskeletal disorders found in different occupational groups such as dentists—48% [2], nurses—45.8% [3], telephone operators—43.2% [4] and office workers—63% [5], among others.

The origin of these musculoskeletal disorders is considered multifactorial [6], with a strong association having been demonstrated between biomechanical risk factors related to posture and movement and the occurrence of work-related neck pain [7,8]. Inadequate postures affect joint kinematics and muscular recruitment, promoting an increase in compressive load on the cervical column and generating pain and disorders in the region [9].

The association between awkward postures and the development of musculoskeletal disorders indicates the need for recording neck posture and movement in occupational settings in order to allow that these factors can be quantified and evaluated [10]. Nevertheless, Ariens *et al.* [6], in a literature review, emphasize a lack in studies evaluating physical exposure using standardized methods of direct measurement of acceptable quality.

Over the last decade, new portable equipment for registering posture and movement in the workplace, such as electrogoniometers and inclinometers, has become available. Initial evaluations of these direct measurement systems have suggested that they are both precise and reliable [11–13]. Other desirable characteristics are that they can be easily operated and don’t interfere with work tasks [14–17]. Furthermore, they should allow for evaluations of all neck movements during the whole shift work and be sensitive enough to identify small variations in movement.

Therefore, the objective of this literature review was to investigate the applications and limitations of the systems for direct measurement of neck movement in the workplace. To this end methodologically qualified studies were identified and evaluated regarding the types of neck movement recorded, the occupational groups evaluated and the principal results obtained.

## 2. Methods

### 2.1. Literature search strategies

A search of the databases Cochrane Library, Cinahl, Embase, Lilacs, PEDro, Pubmed/Medline and Web of Science/Science Direct was conducted using the following keywords: neck, cervical spine, head, posture, movement, risk factors, work exposure, occupational exposure, work related musculoskeletal disorders, pain, symptom, discomfort, recording, workplace, worksite, work, job and occupational activity. Each electronic database was searched to identify studies published in English from the first available year until June, 2009.

#### Inclusion criteria

In order to be accepted for this review, the presence of the following three aspects was required: the use of *direct measurements* of posture and/or movement of the *neck* of active workers in their *occupational settings.*

##### Exclusion criteria

All studies that did not simultaneously address the above-mentioned aspects were excluded from this review.

### 2.2. Procedures for the identification of studies

Initially, two independent reviewers selected studies based on their titles, excluding those that were clearly not related with the theme of the review. Subsequently, the abstracts of all selected titles were analyzed to identify those that met the criteria of inclusion. The potentially relevant articles were obtained in full version for final evaluation. The reference lists of these articles were checked independently by the two reviewers to identify potentially relevant studies that might not have been found in the electronic search. Any disagreements during the process were discussed until a consensus was reached.

### 2.3. Procedures for the evaluation of studies

The two reviewers independently evaluated the methodological quality of the studies using an adapted list of criteria (Table 1) from the one proposed by Ariens *et al.* [6] for evaluating the methodological quality of observational studies.

This list assesses studies regarding their validity and precision, and includes the following categories: study objectives, population studied, exposure measurements, result measurements, and analysis of data. Since the objective of this review was to evaluate the methodological quality of studies regarding physical measurements of occupational exposure, the items in Ariens *et al.* [6] that were not highly associated with the quality of direct measurements were not considered, such as psychosocial factors. Besides, only cross-sectional studies matched the inclusion criteria of this study. Therefore, the items of criteria list only related to case-control and cohort studies were not evaluated. Table 1 highlights the items that were actually assessed.

### 2.4. Evaluation of methodological quality

The included studies were evaluated according to the adapted scale, receiving either a positive (+) or a negative (−) mark for their treatment of each item in question. Any item for which information was not clearly presented was marked as *not described* (ND). Items classified as positive received one point. Since there were six items included in the scale, the maximum potential score would be six points. Nevertheless, one of the items (Exposure measurements Item F, Table 1) was also part of the inclusion criteria for the study, making its evaluation for methodological quality redundant. Thus, considering the items that required a score, a study could achieve a maximum of five points. Based on this arrangement, studies receiving at least three points (>50%) were categorized as having high methodological quality [6,18].

The methodological quality of each study was classified by two independent reviewers. Any disagreements were discussed until a consensus was reached. When agreement could not be reached, a third reviewer (senior researcher) was consulted to make a final decision.

### 2.5. Data extraction

The reviewers extracted the following information from the articles independently: the name of the equipment used for recording neck posture, the types of movement recorded by the instrument (neck flexion-extension, lateral flexion and rotation), the duration of postural recording, the objective of the study, the number of subjects evaluated, the occupational activity evaluated and the numerical results regarding posture or neck movements.

### 2.6. Levels of evidence

Point systems for levels of association between risk and development of musculoskeletal disorders are generally used in reviews of cohort, case-control and cross-sectional observational studies in the workplace [6,18]. Nevertheless, no such system could be used in this review as there were no cohort or case-control studies associating risks present in the workplace and the development of musculoskeletal disorders that matched the inclusion criteria. Thus, only cross-sectional studies that recorded postures by means of direct measurement in the workplace were included. Within this framework, the included studies analyzed aspects such as comparisons between genders, between symptomatic and asymptomatic individuals. The levels of evidence established for the cross-sectional studies in this review were based on those of Bradford-Hill [19]:

- Strong evidence: Two or more high-quality studies with consistent multivariate results;
- Moderate evidence: One high-quality study or two low-quality studies with consistent multivariate results;
- Limited evidence: One low-quality study or unadjusted results;
- Conflicting evidence: Inconsistent studies of same quality (consistent high quality or consistent low quality).

## 3. Results and Discussion

### 

#### 

##### Electronic search

The electronic search resulted in a total of 33,666 references, of which 8,108 were identified as duplicate titles; thus 25,558 remained available for reviewer analysis. Each reviewer read, independently, all of the titles retrieved, and of these, 1,576 were considered potentially pertinent.

**Figure 1.**
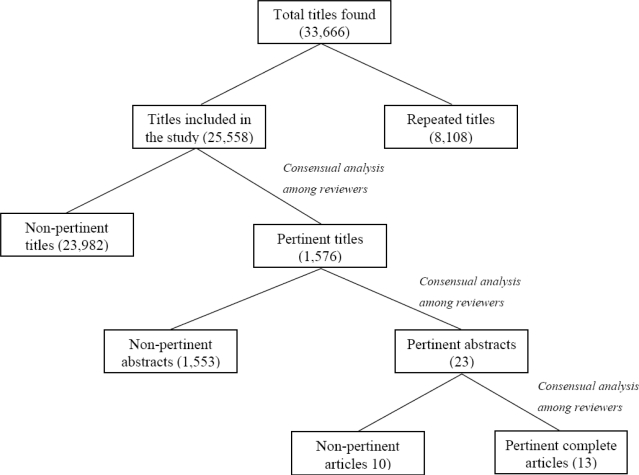
Steps followed for selection of the 13 complete articles included in the study.

The 1,576 abstracts were also read independently by the reviewers and, after new analysis, 23 were considered pertinent to the theme of the review. The complete texts of these studies were located and read. Of these, ten articles were excluded for the following reasons: the methods for using the postural recording equipment were not described, the occupational activities were simulated in laboratories, or workers on leave were included in the study. Therefore, 13 studies were ultimately included in this review. The study selection steps are outlined in Figure 1.

##### Characteristics of the included studies

Table 2 presents the main characteristics of the 13 studies in this review, including: (1) the equipment used for postural recording and the duration of recording, (2) type of neck movement recorded, (3) the objective of the study, (4) occupational activity and number of subjects evaluated and, (5) presented results.

From the data described in Table 2, it was observed that inclinometers were the most common tools for recording neck movement in the workplace. According to Hansson *et al.* [12], this equipment is used to record neck movement because it is practical, portable, and permits long periods of recording in the real work setting. Only three studies used a different type of equipment: two used a physiometer [20,21] and one used an electronic potentiometer [28]. These three studies were published prior to the others.

The recording of neck movement varied between 13 min [27] and 7 h [29], with no association verified between recording time and other aspects of the study.

Regarding the type of movement recorded, neck flexion-extension was evaluated in all included studies. However, although the inclinometers and electronic potentiometers recorded neck lateral flexion movement, only five studies [22,25,28,30,31] reported the results for this movement. Only one study [28] reported neck rotation results from the electronic potentiometer. In part, this could be explained by the equipment used, considering that the measuring principle of inclinometers (the equipment used in 10 of the 13 studies) is the relative angle of the sum-vector of acceleration. In static conditions, this angle coincides with the line of gravity, which makes it impossible to record rotation along the vertical axis [12]. Although inclinometers can record neck lateral flexion, this only occurred in four of the ten studies that used this equipment. This deficiency in the recording of lateral flexion and rotation movements in the neck is a critical aspect as it considerably compromises the understanding of cervical movement. The dynamic of these movements has been recognized as biomechanically and physiologically complex [33,34]. The neck movements occur due to the action of intervertebral discs and the zygo-apophyseal and uncovertebral joints, which represent complementary geometric surfaces. This anatomical configuration determines that movements in the cardinal planes are combined between each other [35–37]. Combination of movements is defined as “the consistent association of one motion around an axis with another motion around a different axis” [38]. Functional neck movements occur around the three movement axes simultaneously. However, it was observed that clinical studies have been investigating each axis of movement separately [39,40]. The combined movements, nevertheless, play an important role in neck functionality [41,42] and are subject to alterations in the presence of pain, lesions and diseases of the cervical column [43].

For this reason, the isolated recording of neck flexion-extension movements by studies in this review does not represent the real postural exposure of individuals in the workplace. Considering the interdependence of cervical movements, any equipment designed to record them should be able to register all movements simultaneously. This will led to the inclusion of simultaneous recordings of the three neck-movement axes in future studies. For this to occur, it would be necessary to either improve the actual systems available or to develop new ones. It is also worth noting that the equipment should not physically restrict neck movement amplitude in any of its axes. Furthermore it should be light, portable and allow for the postural recording during the long periods as the whole work shifts.

Regarding the occupational activity carried out by subjects in the reviewed studies, the recordings were made of workers who performed either sedentary and/or repetitive activities, such as dentists, air traffic controllers and office or industrial workers. The unique study that evaluated the posture and neck movements in more varied activities was Hansson *et al.* [29], which included cleaning workers in its sample. The choice of occupational groups involved in sedentary and repetitive activities could be related to the high prevalence of neck pain complaints in these populations reported in literature [1,44,45]. However, it has also been recognized a high prevalence of neck symptoms in activities considered heavier and more varied, such as, the work of electricians [46] and nurses [3]. Nevertheless, no study on postural exposure evaluated by direct means was located for these jobs.

The purpose for the measurements reported in the studies analyzed here varied widely. The objectives of the studies will be described and discussed together with their methodological characteristics under the heading “Characteristics of the studies associated with their methodological quality.”

##### Evaluation of methodological quality

The results of the methodological evaluation carried out with the adapted scale from Ariens *et al.* [6] are presented in Table 3.

Of the 13 evaluated articles, nine scored ≥3 points and thus were considered to have high methodological quality. Nevertheless, no study got the full score (5 points). A contributing factor to this result was that the item “participation rate” was negative or not described for every study. The strict criterion adopted for a positive mark, which was that at least 80% of the sample had to have been evaluated by direct means, was not accomplished by any of the studies. In some of the studies a large number of subjects were evaluated by means of questionnaires and physical exams, but only a small percentage of these individuals were recorded by direct measurements.

This result demonstrated the difficulty present in studies using direct measurements to evaluate a large number of workers. This is understandable when we consider that the procedures and data analysis for this type of study are highly demanding in terms of data processing and analyzing and are expensive to perform [47]. It should also be taken into account that the worker participation rate will vary considerably when they are invited to either filling out a questionnaire or allowing equipment to be fixed on their body for movement recording during a whole work shift. Thus, the small number of subjects evaluated in studies using direct measurements should be considered a characteristic of this type of study and not a limitation.

Another item that tended to be negatively evaluated by the scale, and for which only three studies [26,30,32] were given a point, was the inclusion of the confidence interval and adequacy of the statistical model used.

Although the majority of studies presented relatively adequate statistical models, they did not describe the confidence interval. The confidence interval has been recognized as advisable for scientific articles as it allow for that inferences can be drawn about the consistency and clinical relevance of the results. According to Sim and Reid [48] this is possible because the confidence interval depends on the variability of the data and the sample size.

##### Characteristics of studies associated with methodological quality

The two studies [23,32] in which gender differences were evaluated were considered studies of high methodological quality. In these two studies, no significant differences were identified between men and women for posture and neck movement during occupational activity, which counts as strong evidence about the subject.

Another two studies of high methodological quality compared symptomatic and asymptomatic subjects [22,25]. In the study by Akesson *et al.* [22], small differences were identified between dentists with and without symptoms for flexion-extension movement of the head and trunk. However, greater differences for the lateral flexion movements of the head and the trunk (26° and 12°, respectively) were reported. Arvidsson *et al.* [25] reported no differences between symptomatic and asymptomatic air traffic controllers for flexion-extension of the head and upper trunk, but in this study the lateral flexion of the head and upper trunk was not numerically reported. These results indicate strong evidence for an absence of difference between individuals with and without symptoms for neck flexion-extension movement. However, there was moderate evidence for the existence of differences between these groups regarding neck lateral flexion movement. These results reinforce the need for evaluating all neck movements simultaneously in studies on the postural exposure of this region of the body.

In two studies of low methodological quality [24,27] and in one of high methodological quality [26], modifications to workstations or in the system of production were evaluated. Arvidsson *et al.* [24] compared the old and new workstations of air traffic controllers and identified a significant reduction in neck flexion after improvements were made to the design. Byström *et al.* [27] evaluated individuals working with computer-aided design (CAD), specifically the two programs PROFESSIONAL-CADAM^®^ and PRO/Engineering^®^, and compared the exclusive use of the mouse to the use of the mouse plus keyboard while operating the above-mentioned programs. The authors reported no differences in worker neck posture and movement during the use of the two programs or during input with the mouse alone and mouse plus keyboard. Balogh *et al.* [26] evaluated the neck overload induced by manual, semi-automatic and automatic systems of production. In this study the authors identified a statistically significant difference between manual and semi-automatic systems, manual and automatic systems, and semi-automatic and automatic systems regarding head flexion. However, all the results considered, no evidence can be reached for these studies evaluating workstation intervention as they investigated very distinct conditions through different clinical outcomes. However, it can be pointed out that the use of direct measurements may be a useful and sensitive resource for identifying variations in posture and movement before and after ergonomic intervention.

Hansson *et al.* [29] and Jonker *et al.* [30] evaluated the correlation between self-reporting of physical overload by workers and the results obtained by direct measurement in two studies of high methodological quality. In both studies correlation between overload reported by workers and the neck angles recorded by inclinometer was not identified. The results of these studies revealed strong evidence for the absence of correlation between these two measuring methods, indicating that one cannot be substituted for the other. Nevertheless, we should consider that these studies were not carried out in situations of more extreme postural exposure, when the perception of individuals tends to become more accurate [49]. Juul-Kristensen *et al.* [31] described the relation between an observational method for evaluating posture and movement and the angles recorded by means of direct measurement. For the observational method, an observer categorized neck flexion as either <20° or >20°. The mean duration of neck flexion >20° was 92% in the observational method and 65% in the inclinometer registration. This difference between methods decreased to 13% after adjustments for the different reference positions. As only one high quality study has compared observational method and direct angle measurements a moderate evidence for differences between these methods was achieved.

Generally, recording protocols consisting of observational methods have the advantage of being inexpensive and practical and can be used in a diverse array of workplaces. Nevertheless, they present limitations such as lower precision when compared to direct measurements, the need for highly trained observers, and restrictions for the use in dynamic tasks, which limit them to more static and repetitive tasks [47,50]. Furthermore, their internal and external validity are questionable [51]. In spite of these limitations, in some occupational situations these are the only possible forms of recording. On the other hand, studies reporting quantitative biomechanics measures taken by direct measurement are complex and, depending on the physical characteristics of the equipment, can influence performance and affect the results [10].

## 4. Final Considerations

The results of this review highlight a lack of studies evaluating the three axes of neck movement simultaneously. This is directly due to deficiencies in the equipment and systems currently available and indicates the need to either the development of new equipment and systems or the improvement of the existing ones. Considering the complexity of cervical movement and the fact that each movement occurring in one plane is necessarily associated with some degree of movement in its orthogonal plane (coupling), the real postural exposure present in occupational activities were not fully recorded so far. That could only be achieved by means of new equipment, which would be able to record the cervical movements simultaneously.

Another deficit identified in the available literature is the lack of studies evaluating the neck posture and movement of workers performing heavier and more varied activities. Considering the high prevalence of neck pain complaints associated with activities, such as, the ones carried out by nurses and electricians [3,46], these studies are still needed.

Moreover, none of the included studies evaluated a sufficient number of subjects by direct measurement to reach the minimum participation rate (80%) required for high methodological quality in studies evaluating occupational exposure [6,18]. This deficiency, however, should be considered with caution. Understanding the methodological difficulties inherent in studies using direct measurement, the small number of evaluated subjects seems to be more a characteristic than a limitation. Thus, specific guidelines for exposure studies are still necessary to assure proper methodological evaluation of these studies.

Finally, this systematic review focused on evaluating the methods of neck movement recording in occupational settings. However, neck posture/movements are only one component of physical load involved in the development of work related neck pain. The force exerted by the hands and the static load in neck region, for example, are also relevant factors related to neck pain and they should be evaluated by valid and reliable methods. Nevertheless, this study has not reviewed the methods of kinetic variables recording which would be important for understanding the quality of kinetic measurements performed in occupational settings.

## Figures and Tables

**Table 1.** Description of the different items in the quality assessment lists proposed by Ariens *et al.* [6]. The highlighted items were applied in this review for evaluating the methodological quality of the studies included.

**Item categories with various definitions**	**Design^a^**			**I, V/P^b^**
*Study purpose*				
A. Positive if a specific, clearly stated purpose was described	Cr	Ca	Pr	I
*Study design*				
B. Positive if the main features (description of sampling frame, distribution by age and gender) of the study population were stated.	Cr	Ca	Pr	I
C. Positive if the participation rate at the beginning of the study was at least 80%	Cr	Ca	Pr	V/P
D. Positive if the cases and referents were drawn from the same population and a clear definition of the cases and referents was stated. Persons with neck pain in the last 90 days had to be excluded from the reference group	Ca			V/P
E. Positive if the response after 1 year of follow-up was at least 80% or if the nonresponse was not selective	Pr			V/P
*Exposure measurements*				
F. Positive if the data on physical load at work were collected and used in the analysis	Cr	Ca	Pr	V/P
G. Positive if the data on physical load at work were collected and used using standardized methods of acceptable quality	Cr	Ca	Pr	V/P
H. Positive if the data on psychosocial factors at work were collected and used in the analysis	Cr	Ca	Pr	V/P
I. Positive if the data on psychosocial factors at work were collected and used using standardized methods of acceptable quality	Cr	Ca	Pr	V/P
J. Positive if the data on physical and psychosocial factors during leisure time were collected and used in the analysis	Cr	Ca	Pr	V/P
K. Positive if the data on historical exposure at work were collected and used in the analysis	Cr	Ca	Pr	V/P
L. Positive if the data on history of neck disorders, gender, and age were collected and used in the analysis	Cr	Ca	Pr	
M. Positive if the exposure assessment was blinded with respect to disease status	Cr	Ca		
N. Positive if exposure was measured in an identical way among the cases and referents	Ca			
O. Positive if the exposure was assessed at a time prior to the occurrence of the outcome	Ca			
*Outcome measurements*				
P. Positive if data on outcome were collected using standardized methods of acceptable quality c	Cr	Ca	Pr	V/P
Q. Positive if incident cases were used (prospective enrollment)	Ca			V/P
R. Positive if the data on outcome were collected for at least 1 year	Pr			V/P
S. Positive if the data on outcome were collected at least every 3 months	Pr			V/P
*Analysis and data presentation*				
T. Positive if the statistical model used was appropriate for the outcome studied and the measures of association estimated with this model were presented (including confidence intervals) d	Cr	Ca	Pr	V/P
U. Positive if the study controlled for confounding factors	Cr	Ca	Pr	V/P
V. Positive if the number of cases in the multivariate analysis was at least 10 times the number of independent variables in the analysis	Cr	Ca	Pr	V/P

a This column shows whether the item was used in the quality list for cross-sectional (Cr), case-referent (Ca) or prospective cohort (Pr) studies.

b This column shows whether the stated item was an information (I) or a validity/precision item.

c This item was scored positive if one of the following criteria was met: (i) for direct measurements, intraclass correlation coefficient >0.60 or kappa >0.40; (ii) for observational methods, intraclass correlation coefficient >0.60 or kappa >0.40; for the inter- or inter-aobserver reliability.

d This item was scored positive if one of the following criteria was met: (i) for self-reported data, intraclass correlation coefficient >0.60 or kappa >0.40; (ii) for registered data, data must show that the registration system was valid and reliable; and (iii) for physical examination, intraclass correlation coefficient >0.60 or kappa 0.40 for the intraobserver reliability.

**Table 2.** Used equipment, duration of the recording, objective of the measurements, occupational activities and relevant findings.

**Article**	**Equipment and duration of Postural Recording**	**Movements recorded**	**Aim of measurements**	Occupational activity and number of workers*	**Relevant Findings**	
Aarås *et al.* [20]	Pendulum Potenciometer (Physiometer) -About 1 hour-	Flexion/extension	To analyze position of the upper arm and head as an indicator of load on the shoulder.	Industrial workers Total: not described Included: 14 workers (11 female, 3 male) Measured: 14 workers	Head flexion was negatively correlated with arm flexion and with load on the upper trapezius muscle.	
Aarås *et al.* [21]	Pendulum Potenciometer (Physiometer) -About 1hour-	Flexion/extension	To study the relationship between postural load for a group of workers and the development of musculoskeletal illness related to length of employment.	Industrial workers Total: 331 workers Included: 331 workers Measured: Not described	Postural load influenced the musculoskeletal sick leave. However, the head flexion influenced the trapezius load much less than the arm position. The workers in redesigned work stations 10C (39–58°) e 11B (15–48°) had greater head flexion than those in original work station 8B (9–31°). In spite of 10C and 11B work stations have lower musculoskeletal sick leave.	
Åkesson *et al.* [22]	Inclinometers (Logger Teknologi) -16 min-	Flexion/extension and lateral flexion	To describe potential neck and upper limb risk factors in female dentists-comparison between symptomatic and asymptomatic workers.	Dentists Total: not described Included: 12 workers Measured:12 workers (6 non-disorders, 6 disorders)	There were not relevant differences between disorders and non-disorders dentists for flexion/extension movements, but higher differences were identified when the lateral flexion movements were analyzed. *Head angles (95th–5th percentile)* 1) Flexion/extension: Non-disorders:41°(7); Disorders: 42°(11) 2) Lateral flexion: Non-disorders:50°(6); Disorders: 24°(7) *Upper back angles (95th–5th percentile)* 1) Flexion/extension: Non-disorders:26°(4); Disorders: 19°(8) 2) Lateral flexion: Non-disorders:25°(7); Disorders: 13°(3)	
Arvidsson *et al.* [23]	Inclinometers (Logger Teknologi) -59min (56–65)-	Flexion/extension	To evaluate the physical workload in a group of women and men.	Air traffic controllers Total: 187 workers Included: 187 workers Measured: 14 workers (7 female, 7 male)	The postural workload showed only minor differences between genders. *Head angles(50^th^percentile)*: Female: 8°(7); Male:12°(6) (p>0.05) *Upper back angles (50^th^percentile)*: Female:13°(12); Male:12°(6) (p>0.05)	
Arvidsson *et al.* [24]	Inclinometers (Logger Teknologi) Old system: 59 min (56–65) New system:51 min (46–55) Break: 40 min (30–49)	Flexion/extension	To evaluate physical exposure, in terms of posture, movements and muscular load among air traffic controllers performing the same work task in two systems.	Air traffic controllers Total: not described Included: 14 workers Measured: 14 workers (7 female, 7 male)	There were large differences in the musculoskeletal loads between old and new systems. During the breaks, the neck ranges were higher than during work. *Neck flexion (95^th^–5^th^percentile)* 1) Female (p<0.05 old vs. new; p<0.05 break vs. work in new and old system) Old:37(4); New:28(10); Break:50(5) 2) Male (p<0.05 break vs. work in new and old system): Old:35(9); New:26(14); Break:50(9)	
Arvidsson *et al.* [25]	Inclinometers (Logger Teknologi) -56 min (36–66)-	Flexion/extension and lateral flexion	To find out whether females with clinically defined neck-shoulder disorders performed this work differently than healthy referents.	Air traffic controllers Total: 70 workers Included: 70 workers Measured: 24 workers (13 cases, 11 referents)	There was no significant difference in neck posture between cases and referents. *Neck flexion/extension (50th percentile)*: Cases:44(9); Referents: 42(10) (p > 0.05) *Neck lateral flexion*: Similar in cases and referents	
Balogh *et al.* [26]	Inclinometers (Logger Teknologi) 1.5 hour (manual) 1hour (semi-automated line) and 4 hours (automated line)	Flexion/extension	To quantify change in physical workload as a consequence of the stepwise technical development of three generations of production system designs.	Operators processing wooden boards for parquet flooring Total:152 workers Included: 152 workers Measured: 31 female operators (25 manual and semi-automated and 6 automated line)	There were evident differences between all three system designs. The automated line showed larger range of motion for the head while the semi-automated line showed the lowest one. *Head angles* (p < 0.05 ^x^manual *vs.* semi-automated, ^y^manual vs. automated, ^z^semi-automated vs. automated) 1) Manual: 10th: 4(1;6)^x,y^; 90th: 29(27;31) ^x^ 2) Semi-automated: 10th: −1(−4;2) ^x,z^; 90th: 21(18;24) ^x,z^ 3) Automated: 10th: −10(−17;−2)^y,z^; 90th: 31(24;38)^z^	
Byström *et al.* [27]	Inclinometers (Logger Teknologi) Drawing table (DT): 1) mouse: 26 min 2) keyboard: 25 min Solid modeling (SM): 1) mouse: 23 min 2) keyboard: 22 min Standing: 13 min	Flexion/extension	To determine the physical workload on neck and upper limb in computer aided design (CAD) work, and to evaluate the impact of two different CAD applications, two different input devices and sitting and standing work positions.	VDU workers Total: 16 workers Included: 15 workers Measured: 9 workers (male)	*DT using a mouse* *Head angle:* 10^th^: 4(−3–15); 90^th^: 21(13–33) *Upper back angle:*10^th^: 5(−13–33);m90^th^: 12(−9–46) *Comparing the applications* The applications did not have a large impact on the postures. The inter-individual differences were bigger for upper back. *Comparing input devices* Non significant differences were found for comparison between devices. *Comparing standing and sitting* Forward head bending was higher when standing and forward upper back lower.	
Eklund *et al.* [28]	Electric Potenciometers (Nickometer,Goteborg) Fork lift trucks: 40 min Forestry machines:30 min Cranes: 40 min	Flexion/extension, lateral flexion and rotation	To identify important causes of postural load for work vehicle drivers, especially head posture.	Work vehicle drivers Total: not described Included: 16 workers Measured:16 workers (3 female, 13 male) 5 fork lift trucks 9 forestry machine 2 crane operators	*Fork lift drivers* Head was twisted to the left when driving, and to the right when handling goods. When high above the ground, head extension occurred in combination with rotation. *Forestry machine drivers* More head rotation occurred using a rotatable cabin than in other machines. *Crane operators* Conventional crane demanded higher trunk flexion, compensated with slight head extension, compared to the redesigned crane, where there was also less lateral flexion of the head.	
Hansson *et al.* [29]	Inclinometers (Logger Teknologi) 3.5 hours (1–7 hours)	Flexion/extension	To evaluate the agreement between questionnaire-assessed and technically measured mechanical exposure to different posture and movements.	Office workers Total: 363 office workers Included: 276 answered the questionnaire Measured: 41 (24 female, 17 male) Cleaners Total: 273 cleaners Included: 218 answered the questionnaire Measured: 41 (41 female, 0 male)	Regarding the postures, there was almost no agreement between questionnaire-assessed and technically measured mechanical exposure within the occupational groups. *Working with the head:* 1) Bent backward: Office workers (k = 0.18); Cleaners (k = 0.18). 2) Bent forward a little: Office workers (k = 0.34); Cleaners (k = 0.24) 3) Bent forward a lot: Office workers (k = −0.07) Cleaners (k = 0.07) *Working with the back:* 1) Bent forward a lot: Office worker (k = −0.06); Cleaners (k = −0.12)	
Jonker *et al.* [30]	Inclinometers (Logger Teknologi) 4 hours	Flexion/extension and lateral flexion	To examine associations between work postures/movements and self-reported workload.	Dentists Total: 73 dentists Included: 24 dentists Measured: 24 dentists	No significant correlation was found between perception of variables in physical demands at work, perception of workload and the neck angles. *Neck angles*	
					Flexion/extension	Lateral flexion
					10th: −12.5(−16;−9)	10th: −9.5(−11.8;−7.1)
					90th: 27.4(24.2;30.5)	90th: 15.4(12.4;18.4)
					*Neck angles* (back/forward) associated with: repetitive movements (r = 0.07,p = 0.75) monotonous working positions (r = 0.01,p = 0.99) uncomfortable working positions (r = −0.21,p = 0.35)	
Juul-Kristensen *et al.* [31]	Inclinometers (Logger Teknologi) 55 min	Flexion/extension and lateral flexion	To compare postures and movements in repetitive poultry processing plant work using a video-based observation method and direct technical measurements.	Workers in poultry processing Total: not described Included: 21 workers (3 workers were excluded due to technical problems) Measured: 18 workers	The difference between the observational method and direct technical measurements was 27% for neck flexion. After adjustments for the different reference positions used, differences in neck flexion decreased to 13%. *Head angles* Flexion/extension: 10th: 8(7); 90th: 31(5) Lateral flexion: 10th: −9(4) 90th: 7(4) *Upper back angles* Flexion/extension 10th: 3(5); 90th: 16(4) Lateral flexion: 10th: −10(4); 90th: 5(4)	
Nordander *et al.* [32]	Inclinometers (Logger Teknologi) 3hours and 58 min	Flexion/extension	To evaluate whether male and female workers performing identical work tasks differ in risk of disorders or in physical or psychosocial exposure.	Repetitive industrial tasks Total: 514 workers Included: 502 workers Measured: 37 workers (19 female and 18 male)	No major gender differences could be found concerning working postures of the head. *Head flexion/extension* Female: 50th: 22(9.8); 90th: 41(9.2) Male: 50th: 24(6.3); 90th: 43(7.5)	

Total = total number of workers; included = number of workers included in the study; measured = number of workers evaluated by direct measurements

**Table 3.** Methodological evaluation of the studies included in this review. As mentioned in Method, data on physical load using standardized methods (direct recording) at work was applied as an inclusion criterion for the present study, and not considered for the total score sum.

	[20]	[21]	[22]	[23]	[24]	[25]	[26]	[27]	[28]	[29]	[30]	[31]	[32]
*Design*													
Participation rate at baseline at least 80% or not selective	ND	ND	ND	−	ND	−	−	−	ND	−	−	ND	−
*Exposure assessment*													
Data on physical load at work collected and used in the analysis	+	+	+	+	+	+	+	+	+	+	+	+	+
Data on physical load collected using standardized methods of acceptable quality	+	+	+	+	+	+	+	+	+	+	+	+	+
*Outcome assessment*													
Data on outcome collected with standardized methods of acceptable quality	+	+	+	+	+	+	+	+	+	+	+	+	+
*Analysis*													
Statistical model appropriate for the outcome studied and a measure of association (including confidence intervals) presented	−	−	−	−	−	−	+	−	ND	−	+	−	+
Number of cases in the multivariate analysis at least 10 times the number of independent variables	−	−	+	+	−	+	+	−	+	+	+	+	+
*Total score*	2/5	2/5	3/5	3/5	2/5	3/5	4/5	2/5	3/5	3/5	4/5	3/5	4/5
